# Persistent monitoring of insect-pests on sticky traps through hierarchical transfer learning and slicing-aided hyper inference

**DOI:** 10.3389/fpls.2024.1484587

**Published:** 2024-11-22

**Authors:** Fateme Fotouhi, Kevin Menke, Aaron Prestholt, Ashish Gupta, Matthew E. Carroll, Hsin-Jung Yang, Edwin J. Skidmore, Matthew O’Neal, Nirav Merchant, Sajal K. Das, Peter Kyveryga, Baskar Ganapathysubramanian, Asheesh K. Singh, Arti Singh, Soumik Sarkar

**Affiliations:** ^1^ Department of Mechanical Engineering, Iowa State University, Ames, IA, United States; ^2^ Department of Computer Science, Iowa State University, Ames, IA, United States; ^3^ Department of Computer Science, Missouri University of Science and Technology, Rolla, MO, United States; ^4^ Iowa Soybean Association, Ankeny, IA, United States; ^5^ Data Science Institute, University of Arizona, Tuscon, AZ, United States; ^6^ Department of Plant Pathology, Entomology and Microbiology, Iowa State University, Ames, IA, United States; ^7^ Department of Agronomy, Iowa State University, Ames, IA, United States

**Keywords:** insect-pest monitoring, yellow sticky traps, deep learning, transfer learning, Edge-IoT cyberinfrastructure

## Abstract

**Introduction:**

Effective monitoring of insect-pests is vital for safeguarding agricultural yields and ensuring food security. Recent advances in computer vision and machine learning have opened up significant possibilities of automated persistent monitoring of insect-pests through reliable detection and counting of insects in setups such as yellow sticky traps. However, this task is fraught with complexities, encompassing challenges such as, laborious dataset annotation, recognizing small insect-pests in low-resolution or distant images, and the intricate variations across insect-pests life stages and species classes.

**Methods:**

To tackle these obstacles, this work investigates combining two solutions, Hierarchical Transfer Learning (HTL) and Slicing-Aided Hyper Inference (SAHI), along with applying a detection model. HTL pioneers a multi-step knowledge transfer paradigm, harnessing intermediary in-domain datasets to facilitate model adaptation. Moreover, slicing-aided hyper inference subdivides images into overlapping patches, conducting independent object detection on each patch before merging outcomes for precise, comprehensive results.

**Results:**

The outcomes underscore the substantial improvement achievable in detection results by integrating a diverse and expansive in-domain dataset within the HTL method, complemented by the utilization of SAHI.

**Discussion:**

We also present a hardware and software infrastructure for deploying such models for real-life applications. Our results can assist researchers and practitioners looking for solutions for insect-pest detection and quantification on yellow sticky traps.

## 1 Introduction

Insect-pests can affect plants by disrupting or interfering with one or more physiological functions that lead to below-normal performance, such as reduced biomass and grain yield. Insect- pests can damage plants in several different ways, by killing plants, which can leave a gap in the crop stand, and the inability of plants to compensate for the open stand (e.g., some boring insects), general stunting caused by metabolic disruption through the nutrient drain or root damage (e.g., aphids, grubs). Moreover, they can kill branches (some species of scale insect can result in branch die-back) or eat inflorescence (e.g., beetles) or plant organs (e.g., boring insects), at-harvest or post-harvest losses (e.g., borers, weevils, etc) (Singh et al., 2021b; Higley, 1986; Pedigo et al., 2021). Insect-pests also cause damage by spreading diseases (Singh et al., 2021a).

Early detection, counting, and constant monitoring of the insects are vital to manage insect pressure in agriculture and reduce the pests’ infestation (Singh and Singh, 2005; Higley, 1986; Stern et al., 1959), as it helps farmers and agricultural professionals monitor and assess the population dynamics of various insect species within their fields. This information is important for making informed decisions about pest control strategies (Sarkar et al., 2023). By tracking the abundance of insects, farmers can identify potential outbreaks early on and take measures to prevent or mitigate crop damage. Moreover, establishing threshold levels helps determine when the insect population reaches a point where action (e.g., pest control) is necessary (Lima et al., 2020). For instance, if insect populations are increasing rapidly or reaching the action threshold, farmers can implement targeted pest control measures, such as applying insecticides or deploying predators, to prevent significant crop losses (Pedigo et al., 2021).

Furthermore, scouting for pests provides valuable data for integrated pest management (IPM) programs. IPM is a sustainable approach that aims to minimize the environmental impact of pest control while maximizing crop yields. Accurate insect counts help IPM practitioners determine the appropriate timing and intensity of pest control interventions reducing the reliance on broad-spectrum insecticides that can harm beneficial insects and lead to insecticide resistance (Cardim Ferreira Lima et al., 2020). Therefore, insect counting is essential in agriculture as it enables farmers to make data-driven decisions, minimize crop damage, and adopt environmentally friendly pest management practices, ultimately contributing to more sustainable and productive farming systems. Manual methods, such as analyzing sticky traps in the field to observe and quantify insects, are time-consuming and labor-intensive tasks and also requires human expertise in accurate pest identification. Therefore, a more automated insect detection and quantification method will be useful for plant researchers and farmers.

Earlier, insect detection was based on their differences in shape, color, pixel intensities, grayscale intensity, and texture analysis (Bauch and Rath, 2005; Huddar et al., 2012; Ghods and Shojaeddini, 2016). More efficient methods are needed to enable accurate and timely monitoring of large crop production areas, which currently demand significant time and labor. In this regard, AI systems, enabled by machine learning (ML) hold great promise for varied phenotyping, for example in disease phenotyping (Rairdin et al., 2022), yield estimation (Riera et al., 2021) and root traits (Jubery et al., 2021; Falk et al., 2020). Similarly, AI/ML is a necessary tool for automatic insect recognition from images and it has led to the development of several automatic monitoring systems (Barbedo, 2020; Li and Yang, 2020; Li et al., 2021; Rustia et al., 2021a; Wang et al., 2020d; Singh et al., 2021a). For instance, a large deep learning model was developed using citizen science data to detect a wide variety of insects (Chiranjeevi et al., 2023) ‘in the wild’ with high robustness Saadati et al. (2023). Apart from such large-scale models, researchers achieved a mean Average Precision (mAP) score of 63.54% using YOLOv3 Redmon and Farhadi (2018) to detect and classify pests in their “Pest24” dataset, which contained 25,378 annotated images of 24 pest species collected using an automatic imaging trap (Wang et al., 2020a, b, d). A multi-stage deep learning method that included object detection, insect vs. non-insect separation, and multi-class insect classification was proposed, achieving an impressive average F1-scores of up to 0.92 (Rustia et al., 2021b). Moreover, an AI-based pest counting method for monitoring the black pine bast scale *(M. thungergianae*) was developed, which reached a counting accuracy of 95%.

However, there are still challenges to address including data collection conditions (Hong et al., 2021) and hence, there is a lack of robust and field-ready insect monitoring systems (SmartProtect, 2022). Many existing studies use datasets with close-up, high-quality images that do not accurately represent the challenging field environments (Cheng et al., 2017; Kasinathan et al., 2021; Nanni et al., 2020; Pattnaik et al., 2020; Wang et al., 2017, 2020b). Building imaging systems automatically capturing high-quality snapshots of individual insects is difficult, especially for small or flying insects (Barbedo, 2020). Therefore, in many instances, a more practical approach is to capture a surface covered with multiple trapped insects using a single image within a sticky trap in the field (Ding and Taylor, 2016; Jiao et al., 2020; Rustia et al., 2021a; Xia et al., 2015; Zhong et al., 2018). Smaller tiles of individual insect images can then be extracted from the full image for further analysis.

Insect monitoring systems often focus on detecting a single pest, overlooking the potential presence of other species that could provide valuable ecosystem information (Ding and Taylor, 2016; Hong et al., 2021; Nazri et al., 2018; Roosjen et al., 2020). A recent study has shown that vision-language foundation models can be leveraged for zero-Shot (without requiring additional model fine-tuning) insect detection (Feuer et al., 2023). Additionally, many AI practitioners fail to apply strict validation procedures, leading to known methodological pitfalls like “data leakage” (Kapoor and Narayanan, 2022). Previous research has demonstrated that model performance can be overestimated when weak validation procedures, such as random data splitting, are used (Kalfas et al., 2021, 2022). Researchers applied different object detectors to localize and classify the insects simultaneously, such as YOLO, R-CNN, and Faster R-CNN (Li et al., 2021; Nieuwenhuizen et al., 2019). They also take advantage of transfer learning and initialize their model using the COCO dataset (Lin et al., 2014). Leveraging Transfer learning, it has been shown that YOLOv4 and YOLOv5 have relatively good performance in detecting five insect species (Verma et al., 2021). Self-supervised learning can also be effective for developing insect detection models (Kar et al., 2023). However, detecting insects on sticky traps using Deep learning (DL) still has some challenges, such as the lack of training data, the small size of insects, the similarity between different insect species, and significant morphological differences among stages in the life cycle of each species of insect, which makes the detection a complex task (Akintayo et al., 2018). Furthermore, some studies focus on datasets with broad insect classes, making classification relatively easier but not representative of more challenging scenarios (Rustia et al., 2021a; Wang et al., 2020d). To overcome these limitations, future research should address the practicality of capturing images in the field, consider the presence of diverse insect species, and implement rigorous validation procedures to ensure accurate and reliable insect monitoring systems.

In this paper, to address some of these challenges, especially the lack of data, we propose a machine learning framework to identify and localize pests on yellow sticky traps using a state-of-the-art object detector in the YOLO series called YOLOv8. On top of using YOLOV8, we leverage two techniques, namely Hierarchical Transfer Learning (HTL) and Slicing-Aided Hyper Inference (SAHI), to alleviate the issues due to smaller training data and small size of the objects of interest. HTL is an advanced version of traditional transfer learning, which leverages knowledge from a larger dataset to improve accuracy when training on a smaller dataset. It involves multiple steps of transfer learning, using intermediate datasets closely related to the target domain, known as in-domain datasets, to enhance the model’s learning process. This iterative approach allows the model to gain insights from datasets that share similarities with the target dataset, leading to significant improvements in performance. To further enhance the accuracy of detecting smaller-sized pests (e.g., an adult Western Corn Rootworm (WCR) Beetle is typically $\frac{1}{4}$ inch long) in images, we implemented the Slicing Aided Hyper Inference (SAHI) (Akyon et al., 2022) method. It enhances the detection of tiny pests in images by dividing the original image into overlapping patches and independently subjecting each patch to object detection, improving overall performance. It also performs a full-inference step to detect larger objects, and then combines the results from both patch-wise and full inference using Non-Maximum Suppression (NMS) to ensure comprehensive and accurate object detection outputs. We report that using HTL instead of vanilla transfer learning, as in previous works, can improve detection accuracy significantly. The further addition of SAHI into our inference framework proves to be a useful strategy for detection of small insects-pests on yellow sticky traps. In addition, our choice of YOLOV8 lets us scale the YOLOv8 up and down to small and large networks and, at the same time, maintain the inference time and accuracy. Additionally, we report that using HTL instead of vanilla transfer learning, as in previous works, can improve detection accuracy significantly. HTL is an advanced version of traditional transfer learning, which leverages knowledge from a larger dataset to improve accuracy when training on a smaller dataset. It involves multiple steps of transfer learning, using intermediate datasets closely related to the target domain, known as in-domain datasets, to enhance the model’s learning process. This iterative approach allows the model to gain insights from datasets that share similarities with the target dataset, leading to significant improvements in performance. To further enhance the accuracy of detecting tiny pests in images, we implemented the Slicing Aided Hyper Inference (SAHI) Akyon et al. (2022) method to enhance the detection of tiny pests in images by dividing the original image into overlapping patches and independently subjecting each patch to object detection, improving overall performance. It also performs a full-inference step to detect larger objects, and then combines the results from both patch-wise and full inference using Non-Maximum Suppression (NMS) to ensure comprehensive and accurate object detection outputs. SAHI proves to be a valuable technique for object detection during inference.

## 2 Materials and methods

### 2.1 Dataset collection, labeling, and preprocessing

We used Unbaited AM yellow sticky traps (hereon referred as YST; Manufacturer: Pherocon) to examine the utility and success of success of our proposed ML approaches to identify and quantify multiple insects. Throughout the growing seasons of 2021 and 2022, we systematically acquired visual data, primarily focusing on beetles, particularly the Western Corn Rootworm (WCR) Beetle (*Diabrotica virgifera virgifera* LeConte), during their adult (winged) life cycle phase. Additionally, we extended our scope to encompass the identification of flies. This comprehensive data compilation was achieved through the strategic deployment of numerous YSTs across agricultural fields used in our research. Yellow sticky traps are routinely used by entomologists and scouts to monitor the presence of insects in greenhouse and field, deployed as a means of attraction and surveillance of pests. The placement of YST in our experiments was conducted approximately 10-12 days before the anticipated emergence of the insects.

**Figure 1:**
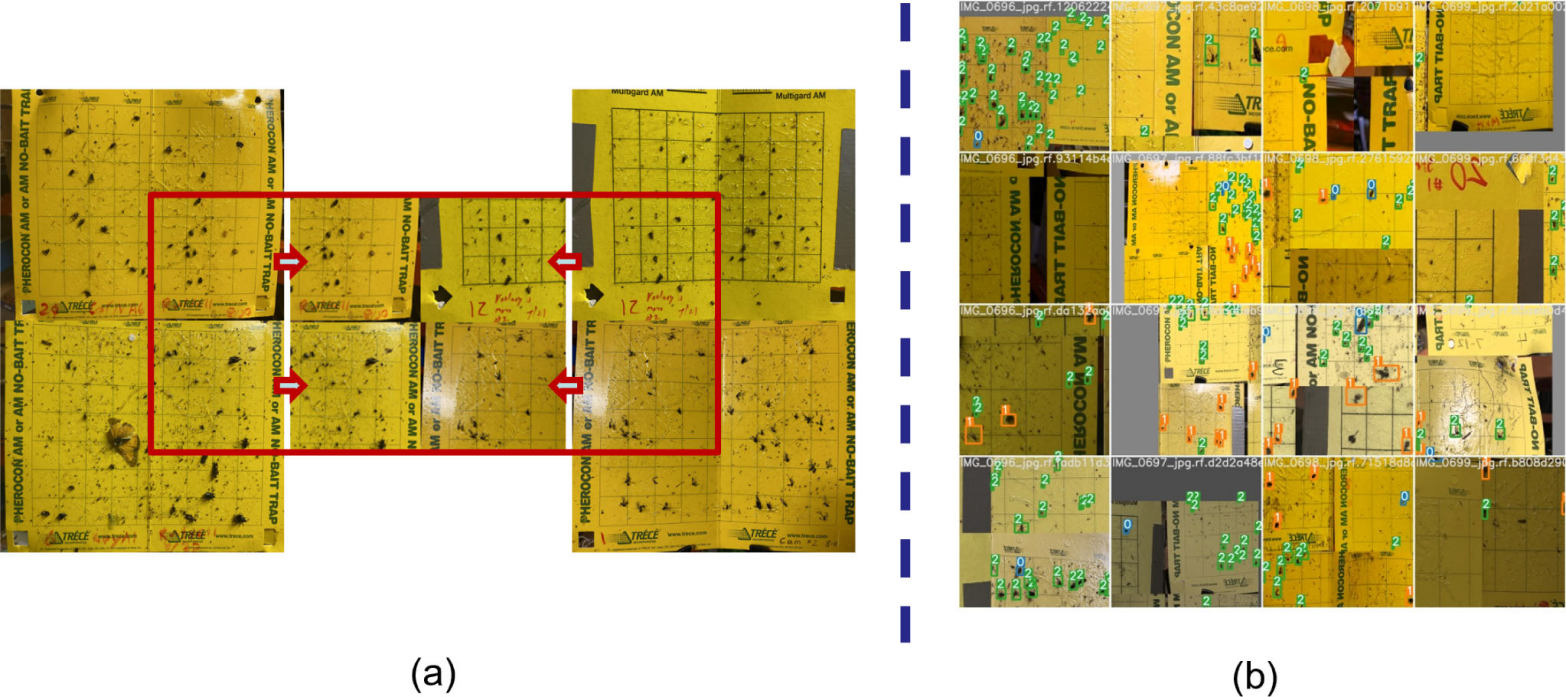
Data augmentation: **(A)** Mosaic augmentation, **(B)** A batch of augmented training data.

The positioning of these YSTs was tailored to the specific target insect. These traps were evenly spaced at intervals of 50 feet, extending from the field’s outer edge to its central region. To ensure their preservation during farming activities such as cultivation and spraying, the traps positioned at the field’s midpoint were distinctly marked. Regular monitoring and inspection of each trap were performed, followed by the capture of trap images using an 8-megapixel camera. These images were subsequently uploaded to a cloud-based server for storage and analysis. Multiple preprocessing and augmentation methods were applied to the data before training the deep learning (DL) model. One notable technique used was mosaic augmentation, which involves creating a single mosaic image by combining slices from four random images in the dataset. This mosaic image is then utilized as a training sample for the model. **Figure 1** illustrates the mosaic augmentation method and showcases the resulting batch of training data after applying all augmentation techniques. We will further explain other preprocessing methods applied in this work in the result section.

### 2.2 In-domain datasets

In addition to our data set described above, we also leverage a few more publicly available insect-pest data sets for training and fine-tuning our model. We refer to them as in-domain datasets and are briefly described below.

#### 2.2.1 Kaggle-Yellow Sticky Traps

The first dataset we consider is The “Yellow Sticky Traps” dataset (Nieuwenhuizen et al., 2019) hosted in Kaggle (hence, referred to as the Kaggle dataset in the Results section). This dataset is centered around addressing the challenges posed by two prominent pests, greenhouse whitefly (*Trialeurodes vaporariorum*) and silverleaf whitefly (*Bemisia tabaci*), which significantly impact greenhouse tomato cultivation in Europe. These insects are among the top 10 most problematic pests in greenhouse vegetable crops. Manually counting and categorizing these insects is time-intensive and prone to errors. Although some automation is introduced through classical thresholding and blob counting algorithms, much of the counting and classification relies on manual effort, sometimes even involving hand counting. This inefficiency hampers effective pest management practices. The dataset’s primary objective is to address this challenge by providing a collection of images captured using yellow sticky traps. This dataset contains 284 images of size 3456 x 5184 and 5184 x 3456. For our use case, we further sliced each image into three pieces to increase the number of images for the fine-tuning task. These images are annotated using the Labelimg tool (LabelImg, 2015), facilitating the identification of distinct classes of insects. Within the dataset, there are three significant classes:

***Macrolophus* MR:** There are 1312 annotations related to *Macrolophus pygmaeus* (MR), a predatory bug commonly employed in biological pest control.

***Nesidiocoris* NC:** This class contains 510 annotations associated with *Nesidiocoris tenuis* (NC), a predatory bug used in integrated pest management strategies.

**Whiteflies WF:** The largest class, with 5591 annotations, corresponds to whiteflies (WF), a significant pest requiring meticulous management. While there is a fourth class (TR) corresponding to Thysanoptera, it has limited annotations and was disregarded.

#### 2.2.2 IP102

The IP102 dataset (Wu et al., 2019) is a meticulously curated collection designed to facilitate insect-pests classification. It undergoes a comprehensive four-stage process, including taxonomic system establishment, image collection, preliminary data filtering, and professional data annotation.

The dataset’s foundation is creating a hierarchical taxonomic system formulated collaboratively with agricultural experts. This system organizes 102 distinct insect-pests classes into a hierarchical structure, wherein each pest is associated with a “super-class” based on the affected crop. The dataset draws from internet resources and employs common search engines and professional websites to gather images and video clips containing insect pests. Extracted snapshots from videos contribute to the comprehensive candidate image collection.

Volunteers trained in insect-pests identification and dataset taxonomy manually review images and eliminate those with irrelevant or multiple pest categories. The selected images are processed, and duplicates or damaged files are removed. Experts are assigned specific crops corresponding to their expertise to accurately categorize the images within the dataset.

In addition to its meticulous creation process, the IP102 dataset boasts significant features. It encompasses over 75,000 images distributed across 102 categories, capturing a diverse and natural long-tailed distribution of insect-pests. This unique characteristic ensures that the dataset accurately reflects real-world occurrences and challenges, making it a valuable resource for advancing research in insect-pests classification and agricultural pest management.

#### 2.2.3 Pest24

The Pest24 dataset (Wang et al., 2020c) is a meticulously curated collection of images capturing various agricultural crop pests to facilitate pest monitoring and detection. For this dataset, 28,958 raw images were taken in 2017 and 2018. These images encompass a diverse array of 38 distinct categories of crop pests from five insect orders: Coleoptera, Homoptera, Hemiptera, Orthoptera, and Lepidoptera. Additionally, they belong to 13 insect families. It is noteworthy that among the mentioned insect orders, the Lepidoptera category stands out, constituting a majority of the 38 field crop pests. Half of these Lepidoptera insects originate from the Noctuidae subfamily. To focus on more prevalent instances, the dataset considers 24 out of the 38 categories as targets for detection, excluding 14 categories with limited instances (ranging from 1 to 11) present in the images.

The dataset refinement process involves the removal of low-quality images. Images exhibiting excessive non-target backgrounds, shadows, occlusions, or inflection spots are filtered out. After this curation, the resulting Pest24 dataset comprises 25,378 annotated images featuring 24 distinct pest categories.

A statistical analysis of the dataset reveals a wide variation in image and object distributions. The most frequently encountered pest in the Pest24 dataset is *Anomala corpulenta*, represented by a substantial 53,347 instances. In contrast, the least frequently present pest is *Holotrichia oblita*, with only 108 instances captured in the images.

### 2.3 Deep learning model

YOLOv8, developed by Ultralytics, represents a recent real-time object detection and image segmentation model that was built upon state-of-the-art advancements in DL and computer vision, delivering excellent speed and accuracy. With its streamlined design, YOLOv8 is incredibly versatile, suitable for various applications, and effortlessly adaptable across various hardware platforms, from edge devices to cloud APIs. One notable aspect of YOLOv8 is its parameter count, which lies between its predecessors YOLOv5 and YOLOv6. It boasts more parameters than YOLOv5 but fewer than YOLOv6. Despite this, YOLOv8 offers approximately 33% higher mAP (mean Average Precision) for various model sizes, consistently outperforming previous versions. The model excels, improving accuracy for different object sizes and types. Furthermore, the inference time with YOLOv8 is significantly faster than any other YOLO model. This efficiency makes it an elegant choice for real-time applications, ensuring that detections can be made swiftly and effectively. Moreover, to cater to different use cases and hardware capabilities, YOLOv8 is available in various model sizes.

### 2.4 Hierarchical transfer learning

Transfer Learning (TL) is a widely recognized approach in ML/DL for harnessing acquired knowledge from one task to improve the performance of a distinct yet related task.

This practice expedites convergence with reduced training data requirement, potentially leading to enhanced generalization capabilities. Recently, the emergence of deep neural networks and the accessibility of extensive pre-trained models have elevated transfer learning to a fundamental instrument across diverse domains.

**Figure 2:**
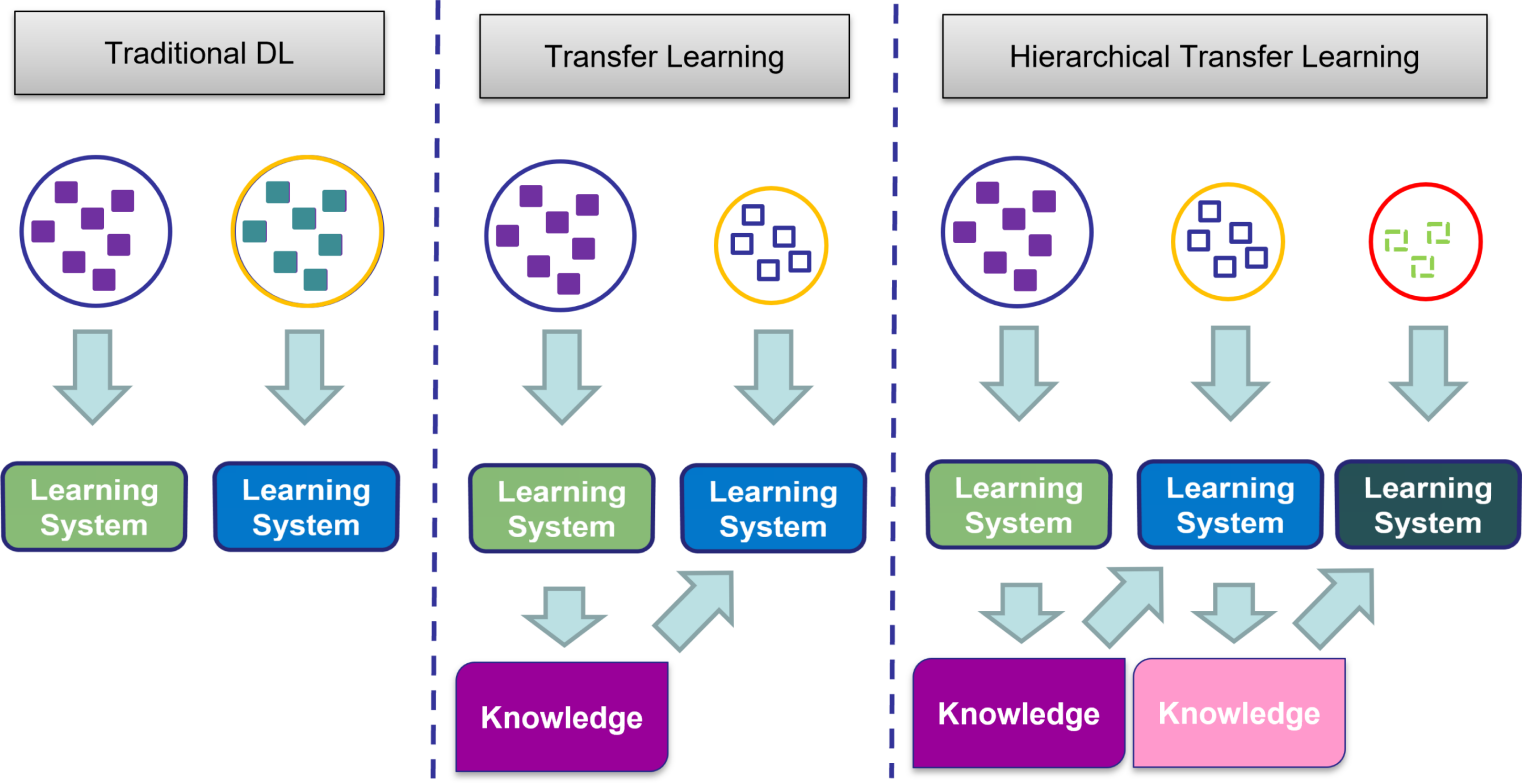
Comparison of traditional learning, transfer learning, and hierarchical transfer learning approaches. Traditional learning involves isolated and independent learning, while transfer learning utilizes knowledge from a previous task for a new task. Hierarchical transfer learning builds upon knowledge acquired from multiple previous learning steps.

HTL extends the foundational concept of transfer learning by incorporating hierarchical frameworks into the process. Instead of directly transplanting knowledge from a pre-trained model to the target task, HTL embraces a multi-step approach where knowledge is gradually transmitted from a source domain to an intermediary domain and subsequently to the target domain. This methodology capitalizes on the notion that certain intermediary domains may share more prevalent features with the target domain, thus facilitating more effective knowledge transfer. **Figure 2** represents the differences between traditional learning, TL, and HTL.

A pivotal advantage of HTL is its capacity to alleviate the negative ramifications of limited data and low resolution. In cases where the available dataset is small, conventional transfer learning methods can still lead to overfitting, as the model heavily relies on the scant available data. HTL mitigates this concern by permitting the model to glean representations from a source domain enriched with more extensive data. Subsequently, the model adapts and fine-tunes these representations to the target domain, which possesses smaller data.

Furthermore, in scenarios featuring diminutive or low-resolution objects of interest, HTL offers notable benefits. Such objects or low-resolution images often lack the intricate details necessary for a direct feature transfer using traditional means. The incremental feature extraction strategy of HTL empowers the model to acquire meaningful higher-level concepts that can be customized to encapsulate crucial attributes of small objects or low-resolution images in the target domain. Due to these reasons, we were motivated to examine the usefulness and applicability of HTL for small object, i.e. insect pests, detection.

### 2.5 Slicing aided hyper inference

To address the challenge of detecting small objects, we employ a versatile framework centered around the concept of slicing during the inference stage. Slicing Aided Hyper Inference (SAHI) is a technique employed during the inference step, and involves the utilization of a “slicing” method to enhance the efficiency of object detection in computer vision tasks. In this method, at first, the original query image, denoted as “*I*” is divided into a number of overlapping patches, represented as “*P*_1_”, “*P*_2_”, and so on up to “*P_l_*”. These patches are formed by segmenting the original image into a grid of smaller sections, each of size “*M* × *N*”. Then each individual patch is then resized while maintaining its original aspect ratio. This resizing step ensures that the patches are suitable for further processing and analysis. Subsequently, object detection is performed independently on each of these overlapping patches. The object detection forward pass involves applying a trained detection model to identify objects of interest within each patch.

**Figure 3:**
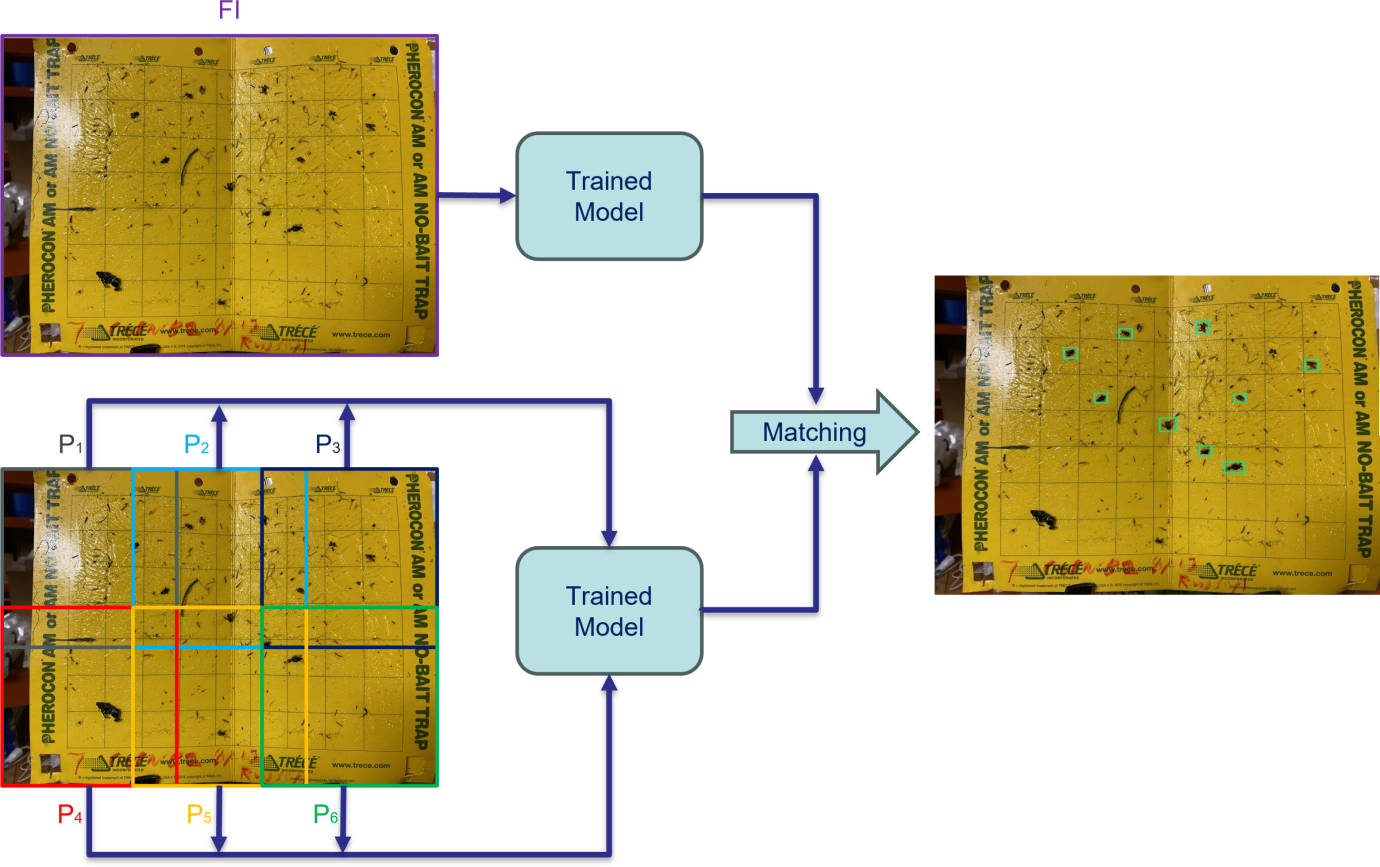
Utilizing slicing-aided hyper inference, the image is divided into overlapping patches $\left(P_{1},P_{2},\ldots \right)$ for individual analysis, alongside Full Inference (FI) for the entire image. The outcomes of patch-wise and FI approaches are merged to produce the ultimate result.

Additionally, there is an optional step called “full-inference” (FI). If opted for, the original, unsliced image can undergo a complete inference process to detect larger objects that may span multiple patches. After the individual patch-based predictions are generated, the results from these overlapping patches, as well as any outcomes from the optional FI step, are combined. This merging process aims to consolidate the detected objects into a coherent output. To avoid redundant and overlapping detections, non-maximum suppression (NMS) is employed. During NMS, detection boxes with higher Intersection over Union (IoU) ratios than a specified matching threshold (*T_m_*) are matched and compared. For each matched pair, detections with a detection probability lower than a specified threshold (*T_d_*) are filtered out and discarded. **Figure 3** shows the schematic of how SAHI was applied for the inference.

#### 2.5.1 Evaluation metrics

Two well-known metrics were used, Intersection over Union (IOU) and Mean Average Precision (mAP) to evaluate our results. IOU can be determined by Equation 1 by considering the ground truth and model-predicted bounding boxes. This metric is used for computing True Positive (*TP*), False Positive (*FP*), and False Negative (*FN*) bounding boxes by considering a special threshold (in this work 0.5).


(1) $$IoU=\frac{\text{Area of Overlap of Predicted and Labeled Bounding Boxes}}{\text{Area of Union of Predicted and Labeled Bounding Boxes}}$$

For calculating mAP, we use the recall and precision metrics as defined in Equation 2.


(2) $$Precision=\frac{TP}{TP+FP},\;Recall=\frac{TP}{TP+FN}$$

Considering the definitions of Precision and Recall, Equation 3 defines Average Precision (*AP*) which is the area under the precision-recall plot for each class.


(3) $$AP=\int_{0}^{1}p\left(r\right)dr$$

The mean average precision (Equation 4) is the mean of *AP*s over a set of queries (*M* is the total number of queries).


(4) $$mAP=\frac{1}{M}\sum_{m=1}^{M}AP\left(q\right)$$

#### 2.5.2 Hyperparameters and evaluation setup

When training an object detection model, a carefully selected set of hyperparameters is crucial for achieving accurate and efficient detection performance. Since our model is used on edge devices the “small” version of the YOLOv8 model architecture was used for this task of insect detection. For training, the initial learning rate is set to 0.01, which will guide the optimization process, while the final learning rate is adjusted to 0.01 times the initial learning rate to determine the rate at which the learning rate will decrease during training. The batch size was set to 16 and “SGD” optimizer were used for training. The choice of momentum at 0.937 and weight decay at 0.0005 helps in stabilizing the training process and preventing overfitting. A warmup period of three epochs is employed at the start of training, with an initial momentum of 0.8 and an initial bias learning rate of 0.1 to gradually transition the model into optimization. Data augmentation is employed to improve the model’s generalization, including HSV-Hue, HSV-Saturation, and HSV-Value adjustments, image rotation, translation, scaling, flipping probabilities (both vertical and horizontal), and mosaic augmentation with a probability of 1.0. To further prevent overfitting, the dropout value was considered as 0.5. The image size for training where considered as 640, and each experiment was trained for 500 epochs. Moreover, The model was trained using a NVIDIA Tesla T4 GPU. These hyperparameters collectively contribute to the training of a YOLOv8 model optimized for accurate and robust object detection.

To evaluate the efficacy of employing HTL, distinct scenarios involving various in-domain datasets were systematically examined. The initial experiment served as a baseline, employing solely pre-trained weights from the COCO dataset. Subsequently, the evaluation extended to encompass the incorporation of specific in-domain datasets - Kaggle (Yellow Sticky Traps), IP102, and Pest24 - in the role of intermediary datasets within the HTL paradigm. This design entailed the successive training of each model on the intermediary dataset, utilizing the trained weights based on COCO dataset. The trained model was subsequently fine-tuned on the distinct Sticky Trap dataset, contributing to a multi-stage/hierarchical training process.

## 3 Results

Before using DL, data preprocessing and augmentation techniques were applied to increase the number of data samples and improve the model’s robustness. In our experimental setup, we employed a rigorous data splitting strategy to ensure the validity and robustness of our results. The dataset was divided into three distinct sets: training, validation, and test. Specifically, we allocated 80% of the data for training, 10% for validation, and 10% for the test set. This test set was kept completely separate and unseen by the model throughout the entire training and fine-tuning process. For training set, each image was tiled to 4 images; therefore, we could increase the data to 628 images. Moreover, before training, techniques such as rotating, zooming, flipping, changing illumination, and mosaic augmentation were applied to the images.

**Figure 4:**
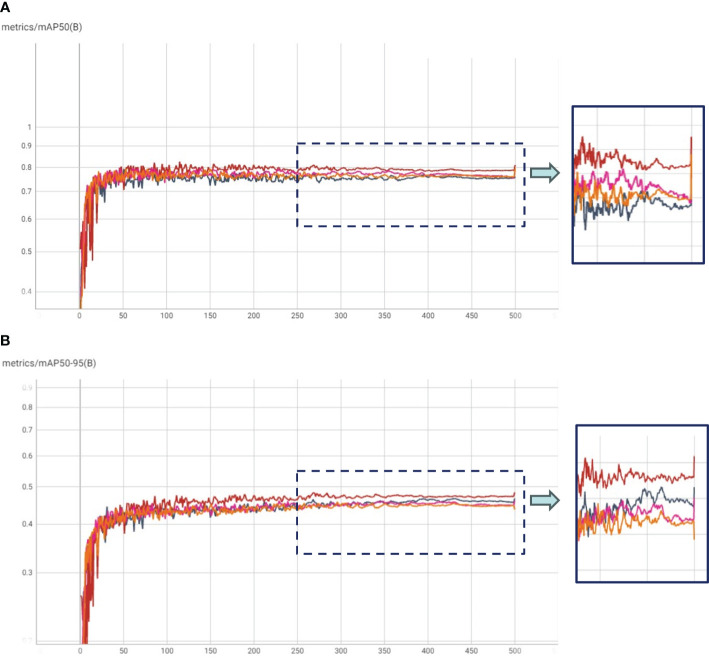
**(A)** mAP50 and **(B)** mAP50-95 plots for Transfer Learning with COCO weights (TL: COCO), and Hierarchical Transfer Learning (HTL) scenarios having Kaggle, IP102 and Pest24 datasets as in-domain datasets.

**Figure 5:**
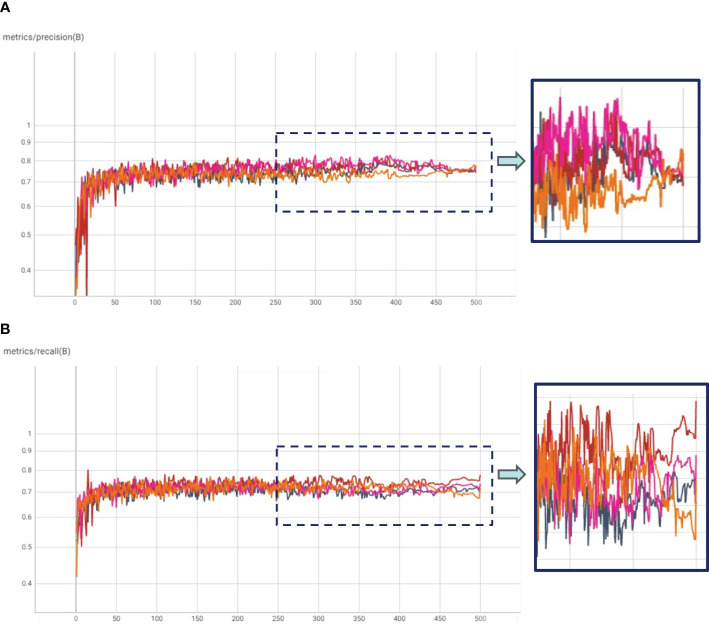
**(A)** Precision and **(B)** Recall plots for Transfer Learning with COCO weights (TL: COCO), and Hierarchical Transfer Learning (HTL) scenarios having Kaggle, IP102 and Pest24 datasets as in-domain datasets.

In our experimental framework, we orchestrated HTL trials, which comprised a two-step fine-tuning process. Initially, the COCO pre-trained model underwent fine-tuning on in-domain datasets, followed by a subsequent fine-tuning phase on our specific dataset. This design yielded three distinct experiment categories: (1) HTL: COCO-Kaggle, (2) HTL: COCO-IP102, and (3) COCO-Pest24, with the Kaggle (Yellow Sticky Traps), IP102, and Pest24 datasets respectively. The results, graphically illustrated in **Figure 4**, unveil compelling insights through mAP and mAP50-95 plots. Notably, the COCO-IP102 experiment emerged as the most successful, excelling in both mAP50 and mAP50-95. This accomplishment can be attributed to the expansive diversity of insect species encapsulated within the IP102 dataset (contains >75,000 images belongs to 102 different insects), encompassing pivotal categories including beetles that align with our focus. While the Kaggle dataset’s limited size hindered it from surpassing Pest24 and IP102, its performance, as depicted in **Figure 4B**, showcased improvements over the baseline in terms of mAP50-95. We posit that a larger Kaggle dataset, given its close resemblance to our data, could potentially yield enhanced results. Despite Pest24’s abundant data, its divergent background and data characteristics pose challenges, thereby compromising insect details, particularly in comparison to the more distinct IP102 dataset. Furthermore, **Figure 5** underscores precision and recall values. These results corroborate the important role played by IP102 as an in-domain dataset, wielding a marked influence in elevating precision and recall metrics. The discernible impact of in-domain datasets on precision is evidenced, reaffirming their role in augmenting overall performance.

**Figure 6:**
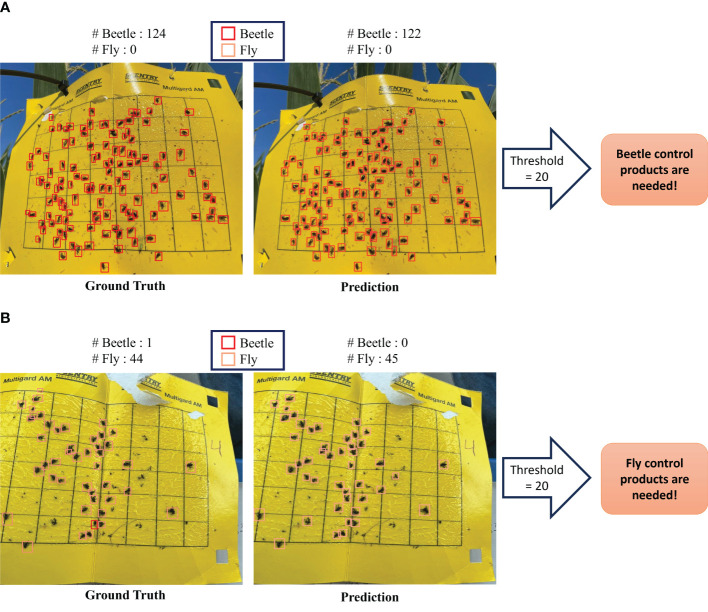
Comparison of detection model outcomes within our framework against ground truth for sticky trap data. **(A)** Example result indicating the need for beetle control products. **(B)** Example result indicating the need for fly control products.

**Figure 7:**
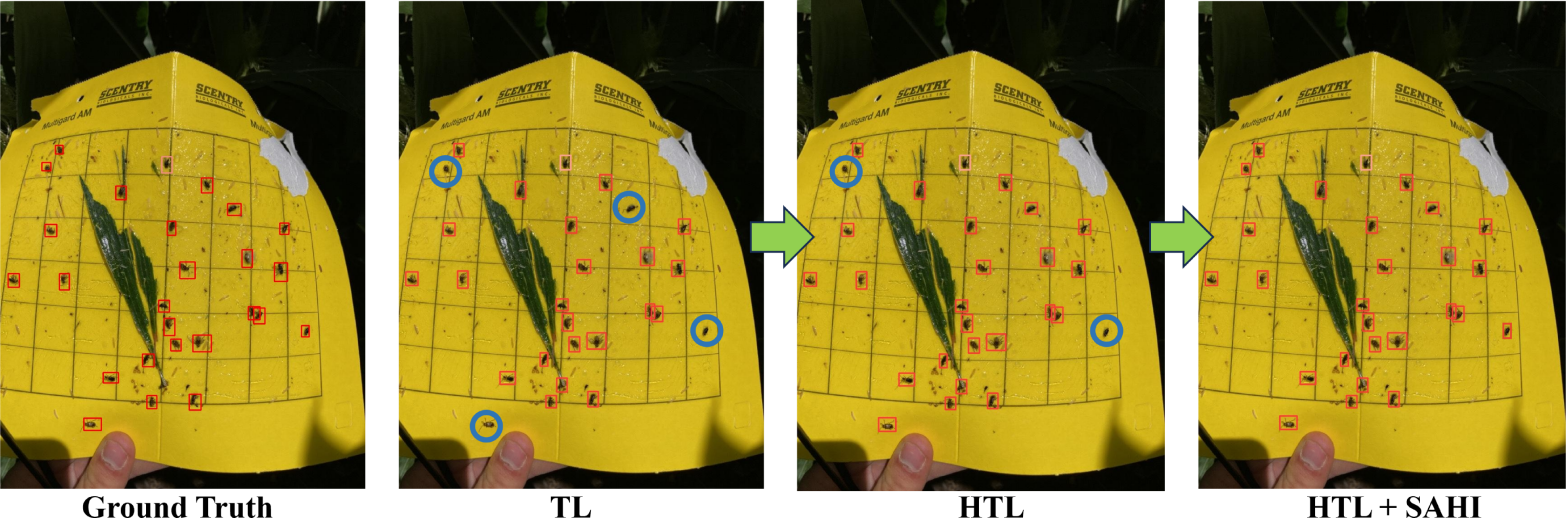
Enhancing small insect detection through HTL and SAHI integration (missed insects are circled).

The practical implications of our model’s performance are shown in **Figure 6**, where the insect detection results are visually presented for two sample images. The result shows the model’s capability to detect small insects through the comprehensive synergy of HTL and the SAHI framework as shown in **Table 1**. These outcomes were obtained from the HTL: COCO-IP102 experiment, which showcases the performance of the approach in the realm of HTL. **Figure 7** illustrates the procedural enhancements facilitated by our method, which incorporates HTL and SAHI, aiming to enhance the detection of small insects.

**Table 1:** The performance of the best-trained model after applying the SAHI method.

Experiments	mAP50	mAP50-95	Precision	Recall	Inference Time
HTL: COCO-IP102	0.82	0.48	0.80	0.78	0.06s
HTL: COCO-IP102 + SAHI	0.86	0.51	0.84	0.83	0.7s

Additionally, in **Table 1**, the inference time for the model on the system with an Intel Core i7 processor and an NVIDIA T4 GPU averages around 0.06 seconds per image (640x640 resolution), with fluctuations ranging from 0.04 to 0.1 seconds. When incorporating the SAHI post-processing method, the inference time increases to an average of 0.7 seconds per image, ranging from 0.3 to 1.2 seconds depending on object density. On a Raspberry Pi 4B, equipped with a 1.8 GHz quad-core Cortex-A72 CPU and up to 8GB of RAM, the inference time, including post-processing, ranges from 8 to 15 seconds per image.

Recognizing the critical importance of insect pressure, we have conducted a comprehensive analysis to determine the insect populations in both predicted and ground truth scenarios. Our findings indicate that, when evaluated against the defined threshold for insect pressure, the predicted results closely align with the ground truth data. This alignment suggests that our predictive model can effectively identify the severity of insect presence, mirroring the accuracy of the ground truth measurements. This threshold serves as a valuable indicator for farmers, enabling them to make informed decisions about the application of insecticides when insect pressure surpasses the established threshold limit. It is worth noting sticky traps were put in the fields with the growing plants; we noted that other than insect-pests, plant parts were also stuck in the sticky traps (see **Figure 7**). Irrespective of this problem, the model could detect the majority of insects compared to the ground truth, thereby demonstrating its usefulness to plant scientists and farmer communities.

### 3.1 Deployment

Our methods provide opportunities for small insect detection; however, this work used digital images from proximity. With advances in ground robots for scouting (Gao et al., 2018) and drone for phenotyping (Guo et al., 2021; Herr et al., 2023), there are significant possibilities for future applications. For example, drones equipped with high-resolution cameras can capture aerial imagery, allowing for early detection of pest hotspots. Meanwhile, ground-based robots can traverse fields using GPS guidance, collecting data on insect presence, activity, and crop health. In addition to advances in phenotyping platforms, there is substantial progress in sensing tools (Sarkar et al., 2023). By deploying these automated scouts, farmers can make data-driven decisions, implement targeted interventions, and minimize the use of insecticides, thereby promoting sustainable and environmentally friendly farming practices in the quest for increased crop yields and food security. We show the combination of ML (HTL) and Vision library (SAHI) can solve small insect detection problems. These innovative technologies provide farmers with a swift and efficient means of surveying vast fields and identifying potential infestations. We provide a description of our hardware and cyberinfrastructure setup below for efficient deployment of the proposed system.

#### 3.1.1 Hardware setup

**Figure 8:**
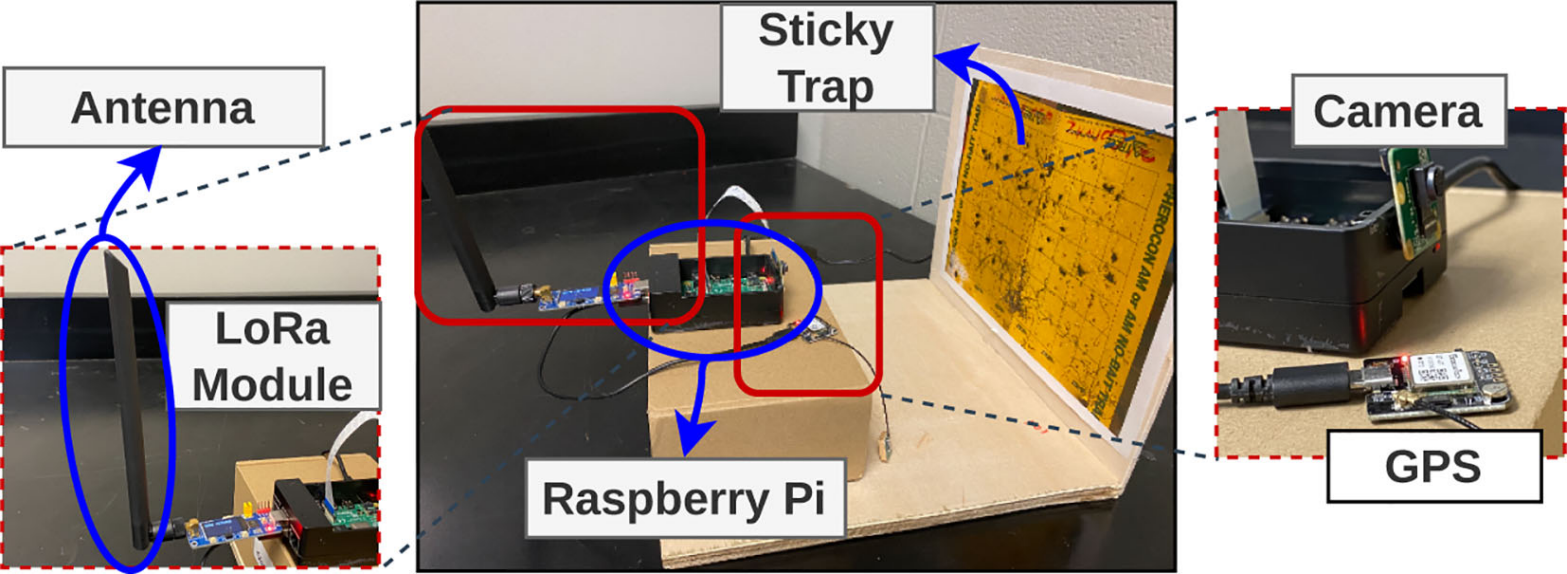
We have developed a prototype that captures images and utilizes a trained model on a Raspberry Pi for insect detection, and obtaining the results in real-time. These results are then transferred to another farm or edge device using a LoRa module (REF).

Following the completion of our training phase and in readiness to implement our insect detection model in real-world scenarios, we created eight distinct prototypes of the sticky trap setup. Each prototype has been meticulously equipped with various necessary components, ensuring seamless functionality and performance. These components include a Raspberry Pi 4B with a robust 8GB RAM capacity, an advanced 8MP camera (Arducam IMX219), ample storage capability of 64GB, a carefully crafted wooden sticky trap holder, an integrated GPS module, and a cutting-edge LoRa communication module. The hardware setup is shown in **Figure 8**. In our initial implementation phase, these prototypes are effectively interconnected with a personal computer, which functions as a computing unit within the Smart Connected Farm (Singh et al., 2023). This connection is established via a local area network, enabling seamless communication and data exchange. For real-time inference validation, we have integrated a pre-trained model. Furthermore, we have established a dedicated web portal designed to facilitate convenient access to the prototypes and to facilitate on-demand image capture from any remote location via the Internet. Presently, access to this portal is facilitated through a virtual private network (VPN). However, we plan to transition the portal to the public domain, ensuring wider accessibility in the near future. This user-friendly interface is an integral part of our proof-of-concept, enhancing the overall functionality and usability of the sticky trap system.

#### 3.1.2 Cyberinfrastructure

**Figure 9:**
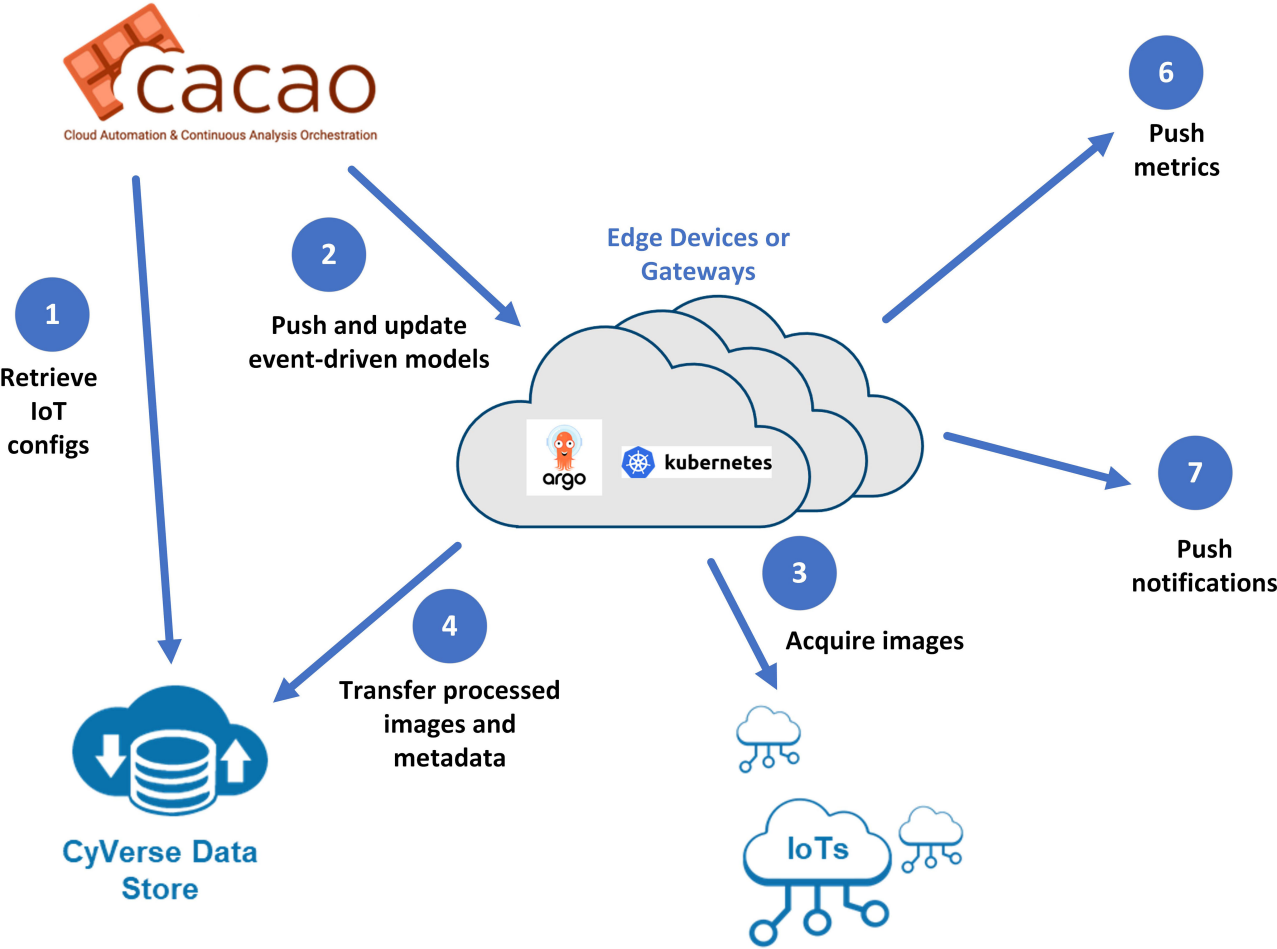
Communication workflow for EDDIE: 1. CyVerse CACAO retrieves configuration information, including models. 2. Models and configurations are deployed to the edge. 3. Images and metadata are streamed to the edge. 4. Images are processed at the edge. 5. Raw and processed data are sent to the central cloud (CyVerse Data Store). 6. User-defined alerts trigger notifications. 7. Metrics are transmitted to metrics servers.

In order to deploy a sustainable persistent sticky trap monitoring system, we have developed EDDIE (Event-Driven Detector for IOT and Edge, see **Figure 9**), an integrated edge management platform that connects the MLOps tasks along with data management components. EDDIE aims to address the research challenges of securely deploying models to the edge or IoT devices and managing the ingest of IoT data where there may be limited or intermittent connectivity. It also provides system alerts and triggers for downstream events based on user-defined conditions. Configurable workflows, which may include ETL operations and one or more models, are executed using Argo Workflows (argo, 2024) on a Kubernetes service designed for resource-constrained environments.

EDDIE is designed with security in mind, encrypting communication from the edge to one or more clouds. In its initial deployment, we used the CyVerse Data Store for its ability to upload data in a high throughput fashion, strong metadata features, and encryption capabilities. When an edge gateway or IoT device is initially configured, models are initially pushed to the edge or can be deployed manually on devices. As researchers update the models on the central cloud, EDDIE components on the edge will discover these changes and pull the updated models and configuration utilizing the DVC framework (Kuprieiev, 2024). EDDIE provides the ability to send selected images and metadata from edge devices to storage end points for further examination.

In our current application, the captured image data (of yellow sticky traps) was uploaded to the CyVerse Data Store on successful detection, and the counts and insect types, along with the processor system’s load and performance of ML methods, were recorded in the IoT metrics component of EDDIE. If the threshold of harmful insect count exceeded the allowable level, alerts were posted to multiple external systems (Slack and webhooks of the IoT metrics server). The full platform was deployed and managed through CyVerse CACAO (Cloud Automation and Continuous Analysis Orchestration) (Skidmore et al., 2023).

## 4 Conclusion

In this study, we extensively explored the efficacy of Hierarchical Transfer Learning (HTL) for the detection of insects on sticky traps under the constraint of limited training data. We demonstrate efficacy of HTL using three publicly available in-domain datasets. The HTL experiments underscore the importance of selecting effective in-domain datasets to optimize model performance. The two-step fine-tuning process revealed that the COCO-IP102 dataset, with its extensive diversity and volume significantly outperformed other datasets. This highlights the necessity of choosing datasets that not only align closely with the target application but also encompass a wide variety of classes and scenarios. Although the Kaggle-Yellow Sticky Traps dataset showed promise, its limited size restricted its performance relative to IP102. Conversely, while larger datasets like Pest24 offered abundant data, their less relevant characteristics hindered effective feature extraction. In conclusion, Prioritizing in-domain datasets that reflect the specific conditions and target species of the application, while also ensuring sufficient diversity and volume, is essential for enhancing performance in hierarchical transfer learning. This strategic approach can lead to more robust models capable of achieving superior performance in real-world applications.

To further improve the performance of detecting small sized insects, we consider the Slicing Aided Hyper Inference (SAHI) method; a strategic approach that capitalizes on image resolution that improved the insect detection capabilities. Finally, We present the design of a hardware setup and an efficient cyberinfrastructure for deploying the persistent insect monitoring framework in real life. While our study demonstrates the effectiveness of YOLOv8 combined with HTL and SAHI for insect detection on sticky traps, we acknowledge certain limitations in our approach. Due to the constraints of edge device deployment, which requires careful consideration of memory and computational resources, we were unable to explore more resource-intensive state-of-the-art methods such as transformer-based models. These advanced techniques, while potentially more powerful, are often impractical for deployment on resource-constrained devices. Future research could focus on adapting these models to enhance detection accuracy, should improve performance be a priority.

Sticky trap-based accurate early detection and counting allow for early mitigation of insect pests. This will allow stakeholders to precise control once they know which insect is trapped in a sticky trap and the level of insects based on the counting, allowing better management. The decision to spray insect pests before the pest population reaches economic injury level (EIL) will allow farmers to apply pesticides when the insect population has reached the action threshold. This will prevent using broad-spectrum insecticides indiscriminately, which helps to avoid pest resistance problems as well as leads to sustainable agriculture.

## Data Availability

The datasets presented in this study can be found in online repositories. The names of the repository/repositories and accession number(s) can be found in the article/supplementary material.

## References

[B1] Akintayo A. Tylka G. L. Singh A. K. Ganapathysubramanian B. Singh A. Sarkar S. (2018). A deep learning framework to discern and count microscopic nematode eggs. Sci. Rep. 8, 9145. doi: 10.1038/s41598-018-27272-w 29904135 PMC6002363

[B2] Akyon F. C. Altinuc S. O. Temizel A. (2022). “Slicing aided hyper inference and fine-tuning for small object detection,” in 2022 IEEE International Conference on Image Processing (ICIP). IEEE. Bordeaux, France. 966–970. doi: 10.1109/ICIP46576.2022.9897990

[B3] argo (2024). Argo workflows. Available online at: https://argoproj.github.io/workflows/. (accessed May 15, 2024)

[B4] Barbedo J. G. A. (2020). Detecting and classifying pests in crops using proximal images and machine learning: a review. AI 1, 312–328. doi: 10.3390/ai1020021

[B5] Bauch C. Rath T. (2005). Prototype of a vision based system for measurements of white fly infestation. Acta Hortic. 691, 773–780. doi: 10.17660/ACTAHORTIC.2005.691.95

[B6] Cardim Ferreira Lima M. Damascena de Almeida Leandro M. E. Valero C. Pereira Coronel L. C. Gonçalves Bazzo C. O. (2020). Automatic detection and monitoring of insect pests—a review. Agriculture 10, 161. doi: 10.3390/agriculture10050161

[B7] Cheng X. Zhang Y. Chen Y. Wu Y. Yue Y. (2017). Pest identification via deep residual learning in complex background. Comput. Electron. Agric. 141, 351–356. doi: 10.1016/j.compag.2017.08.005

[B8] Chiranjeevi S. Sadaati M. Deng Z. K. Koushik J. Jubery T. Z. Mueller D.. (2023). Deep learning powered real-time identification of insects using citizen science data. Plant Phenomics. 6, 0170. doi: 10.34133/plantphenomics.0170

[B9] Ding W. Taylor G. (2016). Automatic moth detection from trap images for pest management. Comput. Electron. Agric. 123, 17–28. doi: 10.1016/j.compag.2016.02.003

[B10] Falk K. G. Jubery T. Z. O’Rourke J. A. Singh A. Sarkar S. Ganapathysubramanian B.. (2020). Soybean root system architecture trait study through genotypic, phenotypic, and shape-based clusters. Plant Phenomics. 2020, 1925495. doi: 10.34133/2020/1925495 33313543 PMC7706349

[B11] Feuer B. Joshi A. Cho M. Jani K. Chiranjeevi S. Deng Z. K.. (2023). Zero-shot insect detection via weak language supervision. The Plant Phenome Journal 7 (1), e20107. doi: 10.1002/ppj2.20107

[B12] Gao T. Emadi H. Saha H. Zhang J. Lofquist A. Singh A.. (2018). A novel multirobot system for plant phenotyping. Robotics 7, 61. doi: 10.3390/robotics7040061

[B13] Ghods S. Shojaeddini V. (2016). A novel automated image analysis method for counting the population of whiteflies on leaves of crops. J. Crop Prot. 5, 59–73. doi: 10.18869/modares.jcp.5.1.59

[B14] Guo W. Carroll M. E. Singh A. Swetnam T. L. Merchant N. Sarkar S.. (2021). Uas-based plant phenotyping for research and breeding applications. Plant Phenomics. 2021, 9840192. doi: 10.34133/2021/9840192 34195621 PMC8214361

[B15] Herr A. W. Adak A. Carroll M. E. Elango D. Kar S. Li C.. (2023). Unoccupied aerial systems imagery for phenotyping in cotton, maize, soybean, and wheat breeding. Crop Sci. 63, 1722–1749. doi: 10.1002/csc2.21028

[B16] Higley L. G. (1986). Economic injury levels in theory and practice. Annu. Rev. entomology 31, 341–368. doi: 10.1146/annurev.en.31.010186.002013

[B17] Hong S.-J.. (2021). Automatic pest counting from pheromone trap images using deep learning object detectors for matsucoccus thunbergianae monitoring. Insects 12, 342. doi: 10.3390/insects12040342 33921492 PMC8068825

[B18] Huddar S. R. Gowri S. Keerthana K. Vasanthi S. Rupanagudi S. R. (2012). “Novel algorithm for segmentation and automatic identification of pests on plants using image processing,” in 2012 3rd International Conference on Computing, Communication and Networking Technologies, ICCCNT 2012. IEEE. Coimbatore, India. doi: 10.1109/ICCCNT.2012.6396012

[B19] Jiao L. Dong S. Zhang S. Xie C.Wang H. (2020). Af-rcnn: An anchor-free convolutional neural network for multi-categories agricultural pest detection. Comput. Electron. Agric. 174, 105522. doi: 10.1016/j.compag.2020.105522

[B20] Jubery T. Z. Carley C. N. Singh A. Sarkar S. Ganapathysubramanian B. Singh A. K. (2021). Using machine learning to develop a fully automated soybean nodule acquisition pipeline (snap). Plant Phenomics. 2021, 9834746. doi: 10.34133/2021/9834746 34396150 PMC8343430

[B21] Kalfas I. De Ketelaere B. Saeys W. (2021). Towards in-field insect monitoring based on wingbeat signals: The importance of practice oriented validation strategies. Comput. Electron. Agric. 180, 105849. doi: 10.1016/j.compag.2020.105849

[B22] Kalfas I. De Ketelaere B. Beliën T. Saeys W. (2022). Optical identification of fruitfly species based on their wingbeats using convolutional neural networks. Front. Plant Sci. 13. doi: 10.3389/fpls.2022.812506 PMC9204059 35720527

[B23] Kapoor S. Narayanan A. (2022). Leakage and the reproducibility crisis in ml-based science. 10.1016/j.patter.2023.100804 PMC10499856 37720327

[B24] Kar S. Nagasubramanian K. Elango D. Carroll M. E. Abel C. A. Nair A.. (2023). Selfsupervised learning improves classification of agriculturally important insect pests in plants. Plant Phenome J. 6, e20079. doi: 10.1002/ppj2.20079

[B25] Kasinathan T. Singaraju D. Uyyala S. R. (2021). Insect classification and detection in field crops using modern machine learning techniques. Inf. Process. Agric. 8, 446–457. doi: 10.1016/j.inpa.2020.09.006

[B26] Kuprieiev R. E. A. (2024). Dvc: Data version control - git for data models. Zenodo. Available online at: https://zenodo.org/record/5654595. (accessed October 30, 2021).

[B27] LabelImg (2015). Free software: Mit license. Available online at: https://github.com/tzutalin/labelImg. (accessed November 15, 2022)

[B28] Li W. Zheng T. Yang Z. Li M. Sun C. Yang X. (2021). Classification and detection of insects from field images using deep learning for smart pest management: A systematic review. Ecol. Inf. 66, 101460. doi: 10.1016/j.ecoinf.2021.101460

[B29] Li Y. Yang J. (2020). Few-shot cotton pest recognition and terminal realization. Comput. Electron. Agric. 169, 105240. doi: 10.1016/j.compag.2020.105240

[B30] Lima M. C. F. de Almeida Leandro M. E. D. Valero C. Coronel L. C. P. Bazzo C. O. G. (2020). Automatic detection and monitoring of insect pests—a review. Agriculture 10, 161. doi: 10.3390/agriculture10050161

[B31] Lin T. Y. Maire M. Belongie S. Hays J. Perona P. Ramanan D.. (2014). “Microsoft coco: Common objects in context,” in Lecture Notes in Computer Science (Including Subseries Lecture Notes in Artificial Intelligence and Lecture Notes in Bioinformatics), Springer International Publishing, Cham, Switzerland. vol. 8693. , 740–755. doi: 10.1007/978-3-319-10602-148/COVER

[B32] Nanni L. Maguolo G. Pancino F. (2020). Insect pest image detection and recognition based on bio-inspired methods. Ecol. Inf. 57, 101089. doi: 10.1016/j.ecoinf.2020.101089

[B33] Nazri A. Mazlan N. Muharam F. W. (2018). Penyek: Automated brown planthopper detection from imperfect sticky pad images using deep convolutional neural network. PLoS One 13, e0208501. doi: 10.1371/journal.pone.0208501 30571683 PMC6301652

[B34] Nieuwenhuizen A. Hemming J. Janssen D. Suh H. Bosmans L. Sluydts V.. (2019). Raw data from yellow sticky traps with insects for training of deep learning convolutional neural network for object detection.

[B35] Pattnaik G. Shrivastava V. K. Parvathi K. (2020). Transfer learning-based framework for classification of pest in tomato plants. Appl. Artif. Intell. 34, 981–993. doi: 10.1080/08839514.2020.1792034

[B36] Pedigo L. P. Rice M. E. Krell R. K. (2021). Entomology and pest management (Long Grove, Illinois, USA: Waveland Press).

[B37] Rairdin A. Fotouhi F. Zhang J. Mueller D. S. Ganapathysubramanian B. Singh A. K.. (2022). Deep learning-based phenotyping for genome wide association studies of sudden death syndrome in soybean. Front. Plant Sci. 13, 966244. doi: 10.3389/fpls.2022.966244 36340398 PMC9634489

[B38] Redmon J. Farhadi A. (2018). Yolov3: An incremental improvement. arXiv preprint arXiv:1804.02767.

[B39] Riera L. G. Carroll M. E. Zhang Z. Shook J. M. Ghosal S. Gao T.. (2021). Deep multiview image fusion for soybean yield estimation in breeding applications. Plant Phenomics. 2021, 9846470. doi: 10.34133/2021/9846470 34250507 PMC8240512

[B40] Roosjen P. P. Kellenberger B. Kooistra L. Green D. R. Fahrentrapp J. (2020). Deep learning for automated detection of drosophila suzukii: potential for uav-based monitoring. Pest Manage. Sci. 76, 2994–3002. doi: 10.1002/ps.v76.9 PMC7496713 32246738

[B41] Rustia D. J. A. Chao J.-J. Chiu L. Y. Wu Y. F. Chung J. Y. Hsu J. C.. (2021a). Automatic greenhouse insect pest detection and recognition based on a cascaded deep learning classification method. J. Appl. Entomology 145, 206–222. doi: 10.1111/jen.v145.3

[B42] Rustia D. J. A. Lu C.-Y. Chao J. J. Wu Y. F. Chung J. Y. Hsu J. C.. (2021b). Online semi-supervised learning applied to an automated insect pest monitoring system. Biosyst. Eng. 208, 28–44. doi: 10.1016/j.biosystemseng.2021.05.006

[B43] Saadati M. Balu A. Chiranjeevi S. Jubery T. Z. Singh A. K. Sarkar S.. (2023). Out-of-distribution detection algorithms for robust insect classification. arXiv preprint arXiv:2305.01823. 10.34133/plantphenomics.0170 PMC11065417 38699404

[B44] Sarkar S. Ganapathysubramanian B. Singh A. Fotouhi F. Kar S. Nagasubramanian K.. (2023). Cyber-agricultural systems for crop breeding and sustainable production. Trends Plant Sci. 10.1016/j.tplants.2023.08.001 37648631

[B45] Singh A. Jones S. Ganapathysubramanian B. Sarkar S. Mueller D. Sandhu K.. (2021a). Challenges and opportunities in machine-augmented plant stress phenotyping. Trends Plant Sci. 26, 53–69. doi: 10.1016/J.TPLANTS.2020.07.010 32830044

[B46] Singh D. P. Singh A. (2005). Disease and insect resistance in plants (New York, NY, USA: Science Publishers).

[B47] Singh D. P. Singh A. K. Singh A. (2021b). Plant breeding and cultivar development (Cambridge, MA, USA: Academic Press).

[B48] Singh A. K. Balabaygloo B. J. Bekee B. Blair S. W. Fey S. Fotouhi F.. (2023). Smart connected farms and networked farmers to tackle climate challenges impacting agricultural production. Front. Agronomy, 6, 1410829. doi: 10.3389/fagro.2024.1410829

[B49] Skidmore E. Cosi M. Swetnam T. Merchant N. Xu Z. Choi I.. (2023). Cloud computing for research and education gets a sweet upgrade with cacao. Pract. Exp. Advanced Res. Computing., 251–254. doi: 10.1145/3569951

[B50] SmartProtect (2022). Smartprotect platform. Available online at: https://platform.smartprotect-h2020.eu/en. (accessed July, 1, 2024)

[B51] Smith R. Van den Bosch R. Hagen K. Stern V. (1959). The integration of chemical and biological control of the spotted alfalfa aphid: the integrated control concept. Hilgardia 29, 81–101. doi: 10.3733/hilg.v29n02p081

[B52] Verma S. Tripathi S. Singh A. Ojha M. Saxena R. R. (2021). “Insect detection and identification using yolo algorithms on soybean crop,” in IEEE Region 10 Annual International Conference, Proceedings/TENCON 2021-December. IEEE. Auckland, New Zealand. 272–277. doi: 10.1109/TENCON54134.2021.9707354

[B53] Wang R. Zhang J. Dong W. Yu J. Xie C. Li R.. (2017). A crop pests image classification algorithm based on deep convolutional neural network. Telkomnika 15, 1239–1246. doi: 10.12928/telkomnika.v15i3.5382

[B54] Wang C.-Y. Liao H. Y.M. Wu Y. H. Chen P. Y. Hsieh J. W. Yeh I. H. (2020a). “Cspnet: A new backbone that can enhance learning capability of cnn,” in IEEE Computer Society Conference on Computer Vision and Pattern Recognition Workshops. 1571–1580. doi: 10.1109/CVPRW50498.2020.00203

[B55] Wang J. Li Y. Feng H. Ren L. Du X. Wu J. (2020b). Common pests image recognition based on deep convolutional neural network. Comput. Electron. Agric. 179, 105834. doi: 10.1016/j.compag.2020.105834

[B56] Wang Q. J. Zhang S. Y. Dong S. F. Zhang G. C. Yang J. Li R.. (2020d). Pest24: A large-scale very small object data set of agricultural pests for multi-target detection. Comput. Electron. Agric. 175, 105585. doi: 10.1016/j.compag.2020.105585

[B57] Wang Q.-J. Zhang S.-Y. Dong S.-F. Zhang G.-C. Yang J. Li R.. (2020c). Pest24: A large-scale very small object data set of agricultural pests for multi-target detection. Comput. Electron. Agric. 175, 105585. doi: 10.1016/j.compag.2020.105585

[B58] Wu X. Zhan C. Lai Y.-K. Cheng M.-M. Yang J. (2019). “Ip102: A large-scale benchmark dataset for insect pest recognition,” in Proceedings of the IEEE/CVF conference on computer vision and pattern recognition. 8787–8796.

[B59] Xia C. Chon T. S. Ren Z. Lee J. M. (2015). Automatic identification and counting of small size pests in greenhouse conditions with low computational cost. Ecol. Inf. 29, 139–146. doi: 10.1016/j.ecoinf.2014.09.006

[B60] Zhong Y. Gao J. Lei Q. Zhou Y. (2018). A vision-based counting and recognition system for flying insects in intelligent agriculture. Sensors 18. doi: 10.3390/s18051489 PMC5982143 29747429

